# Assessing Tachydysrhythmia When P-waves Are Present: Challenges and Pitfalls

**DOI:** 10.7759/cureus.107805

**Published:** 2026-04-27

**Authors:** Brandon Stahl, Joshua Goldwag, Gregory Wu

**Affiliations:** 1 Internal Medicine-Pediatrics, Albany Medical Center, Albany, USA; 2 Radiology, Albany Medical Center, Albany, USA; 3 Emergency Medicine, Albany Medical Center, Albany, USA

**Keywords:** cardiology, focal atrial tachycardia, heart palpitations, narrow complex tachycardia, supraventricular tachycardia (svt)

## Abstract

A 69-year-old female presented to the emergency department with a two-hour history of persistent palpitations. An ECG showed a highly regular narrow QRS tachycardia with P waves. She was ultimately diagnosed with focal atrial tachycardia after an adenosine challenge. Focal atrial tachycardia is a regular, narrow complex, supraventricular tachycardic rhythm that is often paroxysmal and self-resolving. When this rhythm persists, it can eventually lead to tachycardia-induced cardiomyopathy. Narrow QRS complex tachycardia has a broad differential that encompasses many benign and dangerous rhythms. This report highlights the challenges and potential diagnostic pitfalls that can occur while trying to diagnose and evaluate tachydysrhythmias with presenting P-waves. We discuss the management strategies and propose a diagnostic algorithm to aid in the diagnosis of tachydysrhythmias.

## Introduction

Focal atrial tachycardia is a regular, narrow QRS complex, supraventricular tachycardic rhythm that is often paroxysmal and self-resolving. When this rhythm persists, it can eventually lead to tachycardia-induced cardiomyopathy. It frequently occurs in otherwise healthy individuals without structural heart disease [1]. The presence of P-waves in focal atrial tachycardia can complicate the diagnostic picture, making it appear similar to other supraventricular tachycardic rhythms. While the exact prevalence is not well known, a study in the European Heart Journal estimated that 10 to 15 percent of patients presenting for supraventricular tachycardia ablation had atrial tachycardia [2].

Narrow complex tachycardia has a broad differential that encompasses many benign and dangerous rhythms. Initial evaluation and management of narrow complex tachycardia should focus on identifying treatable and reversible causes, such as infection or hypoxia [1]. This report describes the case of a 69-year-old female with sudden-onset persistent palpitations and an initial ECG showing a highly regular tachycardia with P-waves. After an adenosine challenge, the patient was ultimately diagnosed with focal atrial tachycardia. We suggest a diagnostic algorithm to aid in the diagnosis of tachydysrhythmias, along with presenting the therapeutic interventions our patient required to correctly establish the etiology of her tachycardia and restore sinus rhythm.

## Case presentation

A 69-year-old female presented to the emergency department with a sudden onset of palpitations, which had persisted for the past two hours. Her past medical history included hypothyroidism and atrial fibrillation following pulmonary vein isolation ablation, for which she was taking apixaban and metoprolol. She had previously been on flecainide but had stopped it one month earlier. 

**Figure 1:**
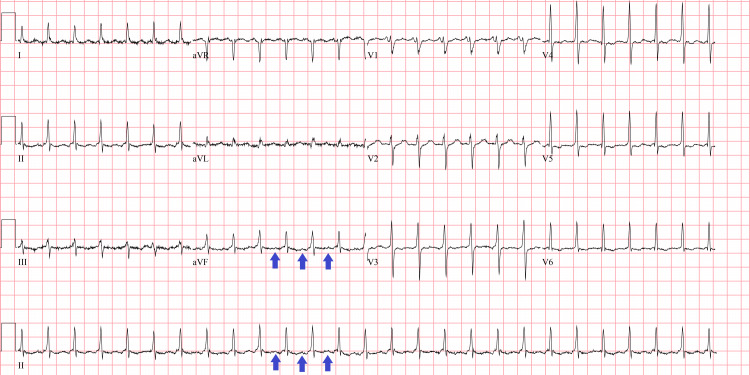
The patient’s initial ECG showing a regular narrow QRS tachycardia with regular P-waves Arrows highlighting P-waves seen in this ECG of regular narrow QRS tachycardia ECG: electrocardiogram

**Figure 2:**
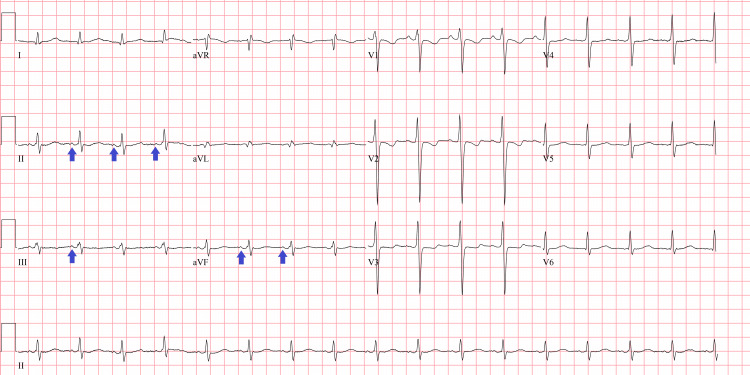
The patient’s ECG from several months prior, showing normal sinus rhythm Arrows highlighting normal P-waves and QRS complexes ECG: electrocardiogram

**Figure 3:**
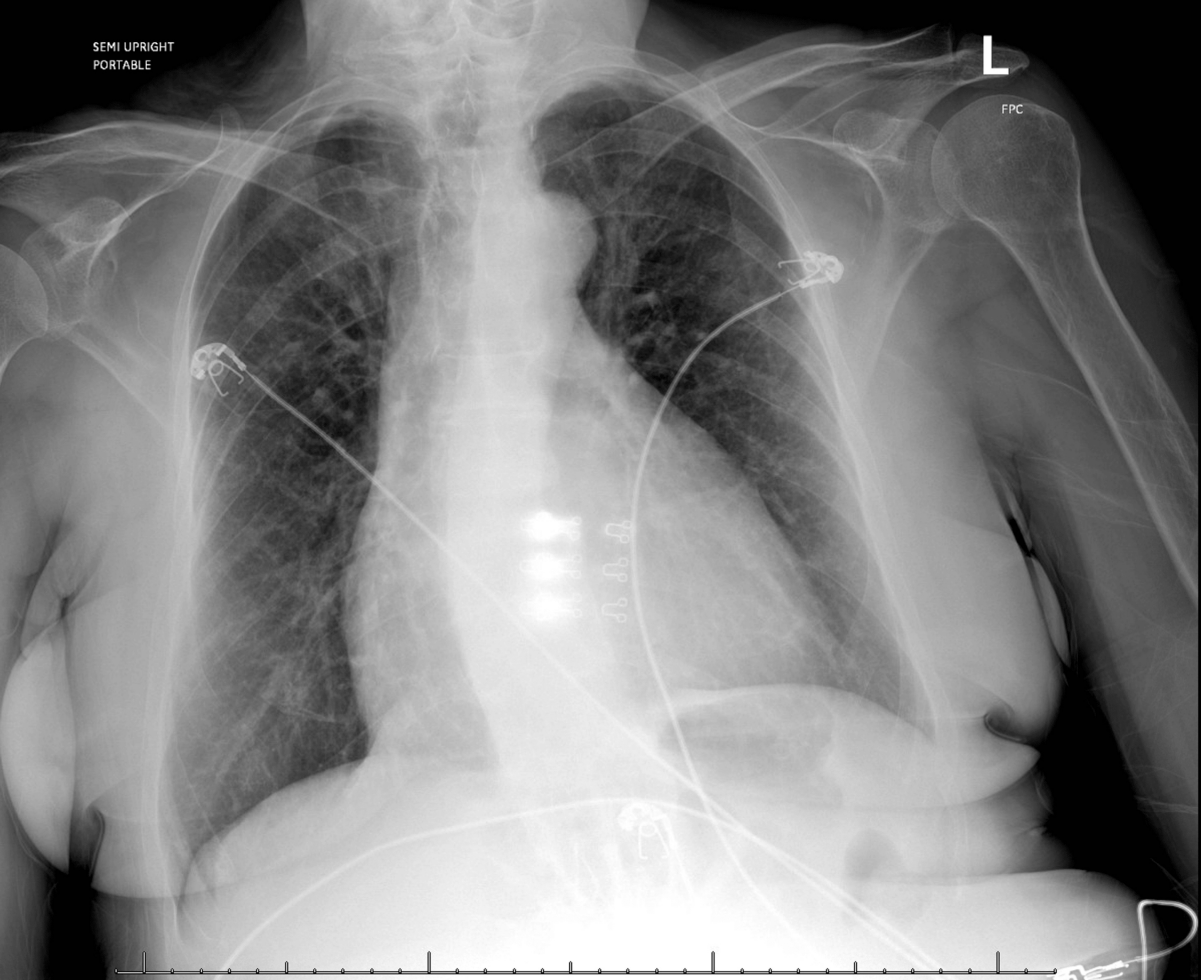
Normal chest X-ray The image depicts a normal chest X-ray without any significant findings

A review of systems was otherwise unremarkable. The patient had taken her morning dose of metoprolol and had taken apixaban before arriving at the hospital. An ECG (Figure 1) was performed and compared with an ECG from a few months earlier (Figure 2). A complete blood count, comprehensive metabolic panel, thyroid-stimulating hormone, free T4, urinalysis, urine culture, magnesium, phosphorus, and high-sensitivity troponin levels were all within normal limits. There was no improvement in the patient’s tachycardia after administration of a fluid bolus. Chest X-ray (Figure 3) was unremarkable, and she did not have any clinical signs of heart failure on exam.

**Figure 4:**
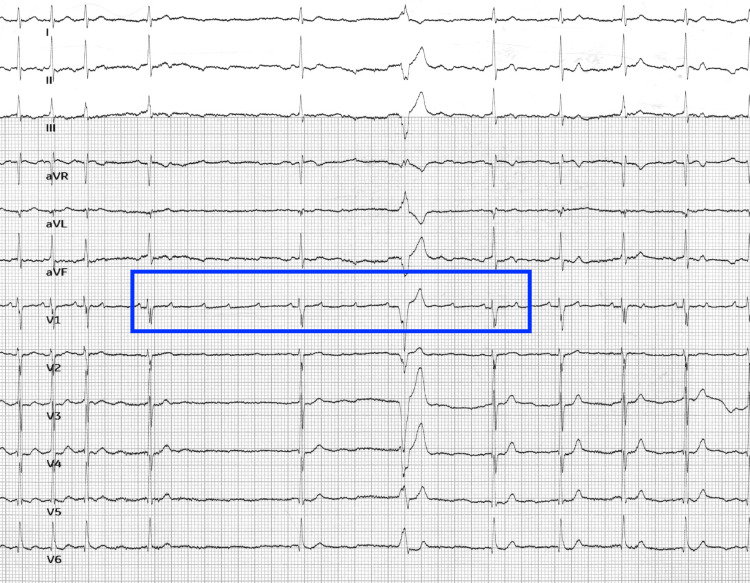
Rhythm strip obtained during an adenosine challenge 6 mg of adenosine was administered, AV nodal blockade achieved after complex 3, exposing a regular P-wave. Blue box highlights the area of highly regular P-waves during adenosine blockade

An adenosine challenge was performed to try to uncover an atrial arrhythmia, such as atrial fibrillation or atrial flutter. A 6 mg dose of adenosine was administered, producing the following rhythm strip (Figure 4), which showed a focal atrial tachycardia.

**Figure 5:**
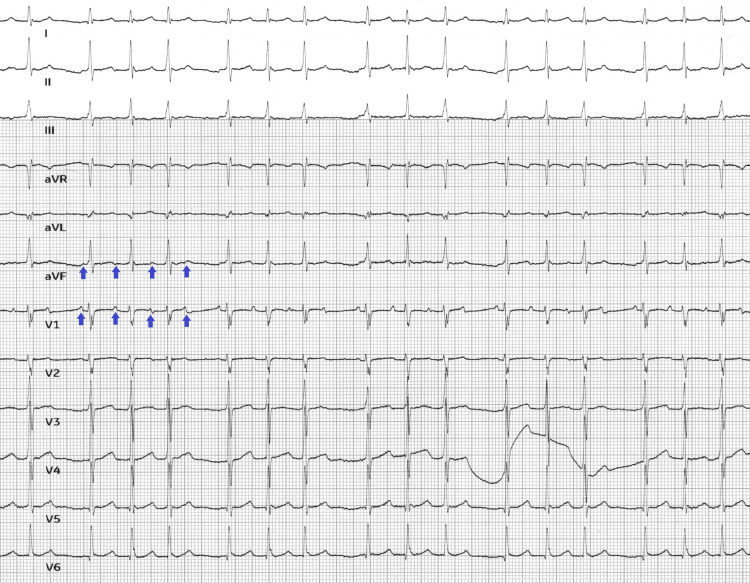
Rhythm strip obtained following administration of metoprolol, diltiazem, and amiodarone The P-wave remains unchanged; however, a Mobitz type I AV block is now present. Arrows indicate progressive prolongation of the PR interval, culminating in a non-conducted P-wave

The patient was given intravenous diltiazem, and subsequent intravenous and oral metoprolol did not produce a response, and she remained tachycardic. She was then given a bolus of amiodarone, and her heart rate decreased to a ventricular rate of 122 beats per minute but showed a Mobitz type I block on ECG (Figure 5). She also became hypotensive, requiring an additional 1 liter bolus of crystalloids. Shortly thereafter, the patient became tachycardic again without evidence of a Mobitz block, which persisted despite amiodarone infusion.

The decision was made to take the patient to the electrophysiology lab, where she underwent sedation and direct current (DC) cardioversion. She successfully cardioverted to sinus rhythm and was discharged the next day.

## Discussion

Focal atrial tachycardia refers to a regular supraventricular tachyarrhythmia caused by a single ectopic electrical focus. Focal atrial tachycardia can arise from micro reentrant circuits and focal ion channel abnormalities. Predisposing factors for focal atrial tachycardia include atrial scarring from ischemic disease, substance use, specifically stimulants and alcohol, digoxin toxicity, and congenital abnormalities. Most cases of focal atrial tachycardia are paroxysmal and self-terminating, but sustained forms can occur and are associated with the development of cardiomyopathy [1]. In the case of adenosine administration, there will be a transient AV block, but the arrhythmia typically does not terminate [3]. Interestingly, our patient was previously on flecainide, a class IC antiarrhythmic that might have been suppressing this arrhythmia, which became apparent after it was discontinued.

**Figure 6:**
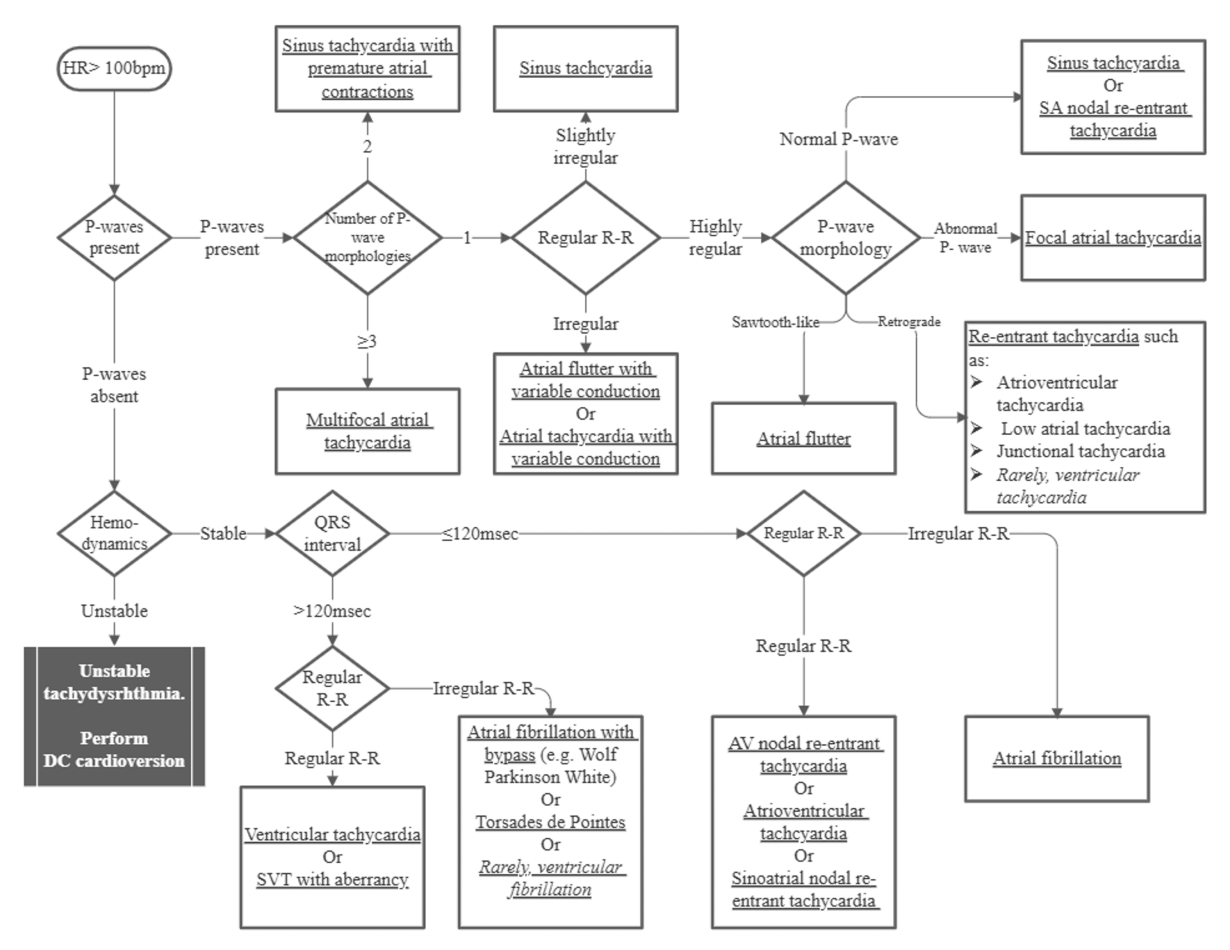
A simplified diagnostic algorithm to assess tachydysrhythmias R-R: R-R interval; SVT: supraventricular tachycardia Figure created by Dr. Wu by using Microsoft Visio

Persistent focal atrial tachycardia can lead to tachycardia-induced cardiomyopathy, which may result in further complications such as dyspnea with exertion, lower extremity edema, and chest pain. Therefore, early and appropriate management of focal atrial tachycardia in these patients is important to avoid these complications [1]. Emergency physicians should not dismiss the presence of a P wave to exclude potentially dangerous tachydysrhythmias. In Figure 6, we present a simplified diagnostic algorithm for assessing tachydysrhythmias in the emergency department based on existing algorithms [4,5].

On ECG, the atrial rate will range between 100 and 250 bpm and can show varying ventricular regularity depending on atrioventricular node conduction. The P wave morphology may be normal or abnormal depending on the location of the ectopic focus, but it will always have consistent morphology from wave to wave; in contrast, multifocal atrial tachycardia will have at least three different P wave morphologies. The Revised 2021 PWM Algorithm is a useful tool to predict the location of the ectopic focus based on the P waves in different leads [6]. For our patient, Figure 1 shows an ECG with supraventricular tachycardia with noticeable P waves. The ventricular rate is highly regular at 159 bpm with narrow QRS complexes. However, the P wave axis is upright in aVR, which is different from the patient’s baseline ECG in Figure 2. An upright P wave in aVR suggests a non-sinus, possibly low atrial or retrograde ectopic focus. An adenosine challenge confirmed the diagnosis, with complete AV blockade showing a monomorphic regular P wave at approximately 150 bpm.

Alternative diagnoses include sinus tachycardia, atrial flutter, atrioventricular nodal reentrant tachycardia, atrioventricular reentrant tachycardia, junctional tachycardia, atrial fibrillation, and multifocal atrial tachycardia. Notably, failure of the arrhythmia to terminate following vagal maneuvers or adenosine administration, even with full AV blockade, is highly indicative of atrial tachycardia, atrial flutter, or sinus tachycardia [3].

## Conclusions

The initial treatment of focal atrial tachycardia should primarily focus on identifying and addressing reversible underlying causes, such as treating ongoing illness, stopping stimulants or alcohol, or adjusting the digoxin dose. Persistent focal atrial tachycardia can be managed with beta blockers or non-dihydropyridine calcium channel blockers such as diltiazem. In the case of refractory focal atrial tachycardia, antiarrhythmic therapy with class IA agents such as procainamide, IC agents such as flecainide, or III agents such as amiodarone can be considered. If medical therapy fails, electrical cardioversion or catheter ablation may be pursued in these patients.
